# Structural engineering of silver nanoparticles for enhanced photoacoustic imaging

**DOI:** 10.1039/d5na00636h

**Published:** 2025-08-21

**Authors:** Rui Zhang, Manuel Dias, Yanchen Li, Stephan Rütten, Fabian Kiessling, Twan Lammers, Roger M. Pallares

**Affiliations:** a Institute for Experimental Molecular Imaging, RWTH Aachen University Hospital Aachen 52074 Germany rmoltopallar@ukaachen.de; b Department of Physics, Faculty of Science, University of Lisbon 1500-274-Lisboa Portugal; c Electron Microscope Facility, RWTH Aachen University Hospital Aachen 52074 Germany

## Abstract

Photoacoustic (PA) imaging is a diagnostic tool widely explored in (pre)clinical settings, as it combines the strengths of optical and ultrasound imaging, resulting in high contrast resolution and deep tissue penetration. Although PA imaging can directly visualize some endogenous molecules (*e.g.* deoxygenated and oxygenated hemoglobin), most of its applications require the administration of external probes, including organic dyes and inorganic nanoparticles. Despite being historically used for antimicrobial and wound healing applications, silver nanoparticles (AgNPs) possess clear merits for PA imaging, including tunable optical properties, high-quality localized surface plasmon (LSP) resonances, strong photothermal conversions, and photostability. In this study, we explored new PA imaging probes based on silver nanocores (with different morphologies and sizes) and polymer shells, and identified the structural features that provide improved biocompatibility, stability, and probe performance. Notably, the size and morphology of the cores strongly impacted the PA signal of the silver probes. For example, among the different particles tested, plate-shaped AgNPs generated up to 3-fold greater signal, as their optical properties, specifically LSP bands and extinction coefficients, were better suited for PA imaging. Even if nanoconstructs displayed apparent inadequate optical features, *e.g.* in the case of spherical AgNPs with LSP bands centered in the blue region of the spectrum, a strong PA signal could still be obtained by manipulating the core size, resulting in up to 2-fold greater signal for larger particles in comparison to their smaller counterparts. All AgNPs were stable in biological environments, did not photobleach, and preserved strong PA imaging signals in *ex vivo* setups. Taken together, our results exemplify the merits of AgNPs as PA imaging agents, providing a better understanding of the nanoengineering of new imaging probes and thereby extending the applications of AgNPs beyond traditional antimicrobial and wound healing applications. Since some of the nanoconstructs we explored in this study are currently being investigated as photothermal agents in clinical trials, new opportunities may arise in intraoperative imaging and image-guided therapy.

## Abstract 2

Our study demonstrates the merits of silver nanoparticles as photoacoustic imaging agents, extending their applications beyond antimicrobial and wound healing.

## Graphical abstract

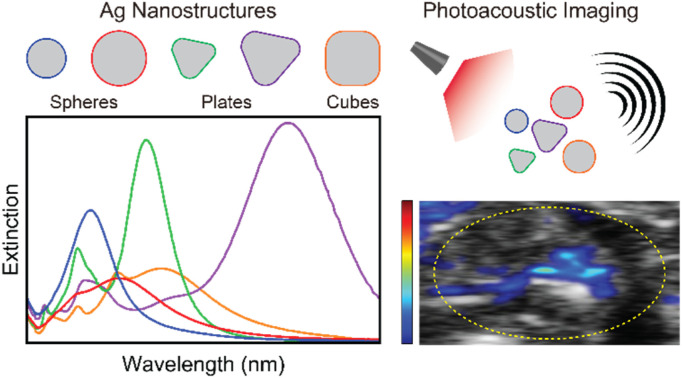

## Introduction

Photoacoustic (PA) or optoacoustic imaging is a non-invasive imaging modality which has been widely explored in preclinical research and has recently been translated into clinical practice.^1–6^ PA signal is generated by irradiated chromophores or probes that absorb the energy of non-ionizing laser pulses and convert it into local heat, generating ultrasound waves.^7,8^ Acoustic signals can provide better resolution than fluorescence, as they are less sensitive to scattering when propagating through tissues.^9,10^ Additionally, some endogenous molecules, such as oxygenated and deoxygenated hemoglobin, possess characteristic PA signals, showing clinical potential to measure blood oxygen saturation as a disease marker.^11,12^ Nevertheless, (pre)clinical PA imaging faces key challenges, including low signal from many natural endogenous chromophores and limited spectral separation between them.^12–14^ To address this gap, near-infrared (NIR) absorbing organic dyes are frequently used as exogenous PA probes;^15–17^ however, they are limited by severe photobleaching, low NIR absorbance, poor light-to-acoustic signal transduction, and insufficient solubility and stability under biological conditions.^13,18,19^ Hence, there is an increasing need for superior exogenous PA probes that provide strong contrast at depth, remain stable during imaging, and are biocompatible.

In the last decade, noble metal (especially gold) nanoparticles have become increasingly adopted as PA imaging probes in preclinical settings because of their unique optoelectronic properties.^20–22^ Noble metal nanoparticles sustain localized surface plasmon (LSP) resonances, which result in strong extinction coefficients and electromagnetic fields at their surfaces.^23,24^ The LSP bands can be shifted towards the NIR by manipulating the size and morphology of the nanoparticles.^25–29^ Moreover, they also display large photothermal conversion coefficients, and their surface can be functionalized with ligands, improving their biocompatibility.^21,24,30^ As a result, anisotropic gold nanoparticles, such as rods and shells, have already moved to clinical trials for photothermal ablation of prostate cancer and atherosclerosis plaque removal,^31,32^ and gold nanostars and rods have been explored preclinically for PA imaging.^20,33,34^ In the case of silver nanoparticles (AgNPs), despite displaying better quality LSP resonances than any other metal nanoparticle,^35^ their uses have been historically limited to antimicrobial and wound healing applications.^36–38^ This has been partially caused by the poor control over their morphology (and optical properties) *via* traditional colloidal methods.^39^ Nevertheless, chemical protocols developed in the last decade have significantly improved the structure manipulation of silver colloids, yielding anisotropic AgNPs with well-defined optoelectronic properties and endowing their use in other applications historically limited to anisotropic gold nanoparticles.^40–42^ As a result, there is a renewed interest in exploring anisotropic AgNPs in photothermal-related applications,^43–45^ such as photothermal therapy and PA imaging, where high-quality LSP can be beneficial for enhanced performance. For example, silver nanoplates have demonstrated excellent performance as PA agents, even under *in vivo* conditions.^46^ Although various silver nanostructure morphologies can be synthetically obtained, a direct comparative analysis of differently shaped nanoconstructs has not yet been performed. Such a study would help to identify the most effective candidates for PA imaging applications.

In this study, we explored the use of five AgNPs made of different silver core structures (plates, cubes and spheres) and sizes (ranging from 50 to 100 nm), and functionalized with a polymeric shell for PA imaging. Plate-shaped AgNPs displayed stronger PA responses than spherical and cubic AgNPs. Both larger-sized silver nanoplates and spherical silver particles presented greater PA contrast abilities compared to their smaller counterparts. The AgNPs preserved their PA performances in complex biological environments as demonstrated by the successful *ex vivo* PA imaging in deceased mice. Our results highlight the capabilities of silver nanomaterials in PA imaging, paving the way for their use in preclinical diagnosis.

## Results and discussion

**Fig. 1:**
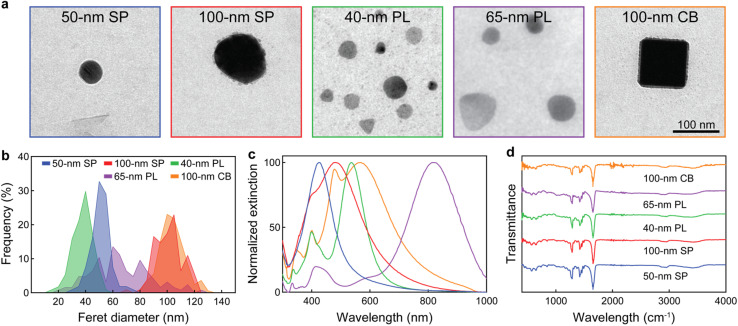
Characterization of the AgNPs. (a) Transmission electron micrographs, (b) Feret diameter distributions, (c) extinction spectra, and (d) FTIR spectra of the silver nanoconstructs.

We explored five different AgNPs made of silver cores and polyvinylpyrrolidone (PVP) shells (Fig. 1a). PVP acted as the stabilizing agent and was chosen because it is a polymer commonly used in drug delivery applications, including in some clinical silver nanomedicines, such as Argovit.^37^ Two nanoconstructs had spherical cores with sizes of 50 and 100 nm (50 nm and 100 nm SP, respectively), as these are sizes commonly used in therapeutic AgNPs.^47,48^ The other three nanoparticles had anisotropic cores, consisting of two particles absorbing at the end of the visible light range, namely 40 nm silver plate (40 nm PL) and 100 nm silver cube (100 nm CB), and one NIR-absorbing 65 nm silver plate (65 nm PL). The latter one is used in the clinical formulation SNA-001, which is currently being explored for photothermal treatments against skin conditions.^37^

The AgNPs were relatively monodisperse, except for the 65 nm PL, which showed a broad range of sizes (Fig. 1b and Table S1). This was consistent with previous studies, which reported that silver nanoplates (particularly those with larger sizes) tend to be highly heterogeneous, due to challenges on limiting their growth to two dimensions and the simultaneous growth of seed-mediated and seedless nanoconstructs.^49–51^ Elemental mapping with energy-dispersive X-ray spectroscopy (EDS) confirmed that all nanoparticles were composed of silver cores (Fig. S1).

Because of their distinct morphologies and sizes, the AgNPs displayed rather different optical properties (Fig. 1c), with LSP band maximum ranging from 426 nm (50 nm SP) to 814 nm (65 nm PL). This nanoconstruct selection allowed to obtain different AgNPs with LSP bands covering the light spectrum from visible to NIR. Notably, 100 nm SP had relatively large extinction in the NIR (despite being spherical) because of its broad LSP band due to its large core size. The presence of PVP on the AgNP surface was confirmed by Fourier-transform infrared (FTIR) spectroscopy, as multiple characteristic PVP peaks were observed (Fig. 1d). For example, the peaks at 1270 and 1420 cm^−1^ were associated with the stretching vibration of C–N, the peak at 1650 cm^−1^ was related to the stretching vibration of C=N, while the peaks at 2890 cm^−1^ and 3400 cm^−1^ were related to the C–H stretching and N–H vibration, respectively. Furthermore, the AgNPs had zeta potentials between −10.6 and −13.6 mV, which were consistent with the ones obtained for metallic nanoparticles coated with PVP,^52,53^ and could be dispersed well in water as indicated by their hydrodynamic diameters (Fig. S2).

**Fig. 2:**
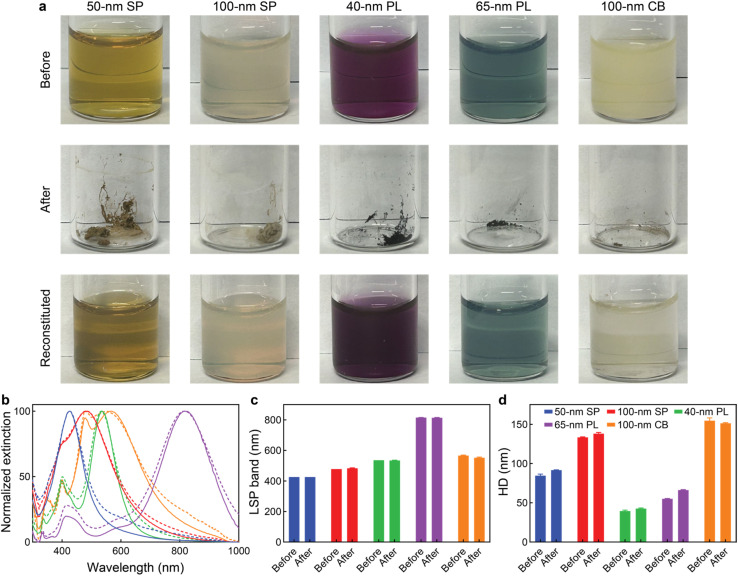
Effect of lyophilization on the AgNP properties. (a) Photographs of the AgNPs before and after lyophilization, and after reconstitution with water. (b) Extinction spectra, (c) LSP band positions, and (d) hydrodynamic diameters (HD) of AgNPs before and after lyophilization. Solid lines represented AgNPs before lyophilization, dashed lines represented AgNPs after lyophilization. Values in columns represent mean ± standard deviation. All measurements were performed in triplicate.

Interestingly, coating the silver cores with PVP had an additional advantage. PVP is commonly used as a cryoprotectant agent during the lyophilization of pharmaceutics and nanomedicines.^54–56^ Lyophilization is a low-temperature dehydration process that removes water by sublimation. This process improves the long-term stability of medicines and decreases their volume and weight, easing their storage and commercialization. The five AgNPs could be lyophilized and resuspended in water without the need of adding any additional cryoprotective reagent, as demonstrated by their optical properties (LSP band position) and hydrodynamic diameters, which were preserved after lyophilization and reconstitution without significant changes (Fig. 2). As expected, for most nanoconstructs, their hydrodynamic diameters were significantly larger than their average core sizes as measured by transmission electron microscopy, since the former include the core sizes and the ligand and hydration shells of the particles. In the case of the silver plates, however, those differences were less pronounced, likely due to their strong anisotropy, since the silver nanoplates were flat (pseudo two dimensional) particles with only 10 nm thickness,^57,58^ rather than three dimensional objects (like spheres or cubes). Notably, all AgNPs displayed good long-term stability, as demonstrated by their unaltered LSP band positions and hydrodynamic diameters during five weeks after reconstitution (Fig. S3).

**Fig. 3:**
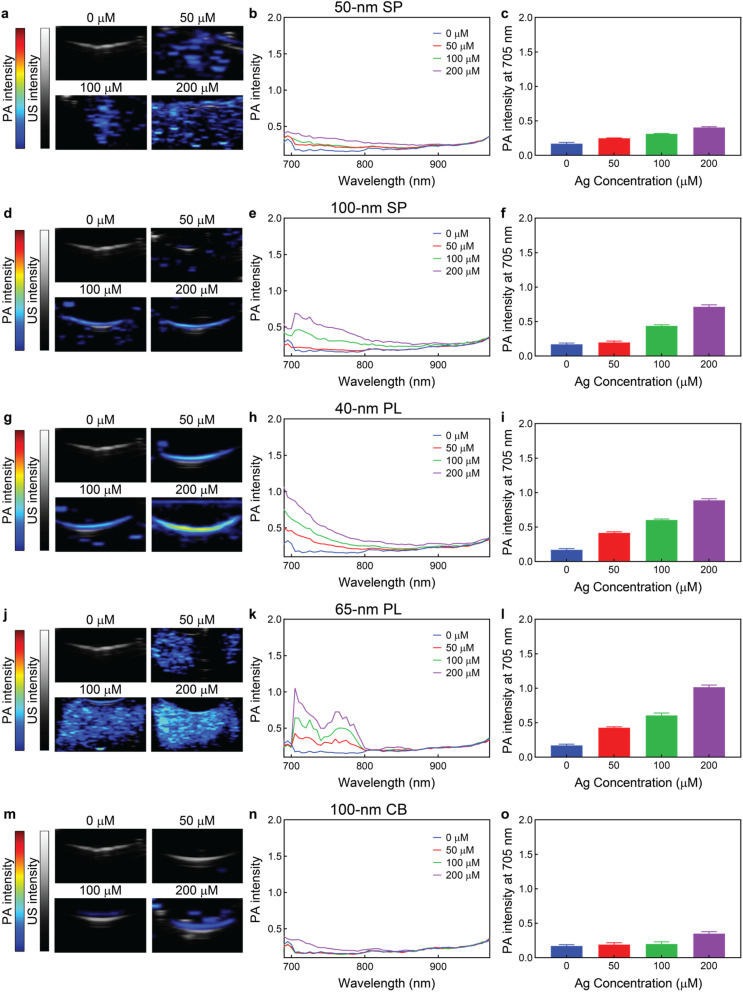
PA-US imaging of the AgNPs in gelatin phantoms. PA-US images, PA spectra, and PA intensities at 705 nm of (a–c) 50 nm SP, (d–f) 100 nm SP, (g–i) 40 nm PL, (j–l) 65 nm PL, and (m–o) 100 nm CB in gelatin phantoms at different silver concentrations (from 0 to 200 μM silver). The scale bar is displayed in linear arbitrary units. Values in columns represent mean ± standard deviation. All measurements were performed in triplicate. All measurements were performed roughly at the same time, and the 0 μM silver samples are the same for the five nanoconstructs.

Next, we evaluated the PA imaging capabilities of the five silver nanoconstructs. The AgNPs were imaged at different concentrations (ranging from 0 to 200 μM silver) in gelatin phantoms with a preclinical PA system that provided both bright-mode ultrasound (US) and PA (from 680 to 970 nm) (Fig. 3). While the 50 nm SP displayed relatively low PA responses (up to 0.40 ± 0.01 at 200 μM silver), which was expected based on their LSP band centered at 426 nm, the 100 nm SP did show considerably greater PA intensities (up to 0.71 ± 0.03 at 200 μM silver). Even though the LSP band of the larger spherical particles was centered at 478 nm, the plasmonic band was very broad due to the large core sizes, providing significant absorption in the NIR region (from 650 to 800 nm), and contributing to the strong PA contrast. Similarly, the 65 nm PL exhibited stronger PA responses than the 40 nm PL (up to 1.01 ± 0.03 and 0.89 ± 0.02, respectively, at 200 μM silver), as the former displayed greater extinction (LSP band maximum at 814 nm and 536 nm, respectively) in the area where the PA spectra were recorded. Interestingly, the 65 nm PL displayed a more spread PA signal in the phantom, which might have been caused by the broader size distribution of the particles compared to other AgNPs (Fig. 1b). Lastly, even though the 100 nm CB LSP band had a certain overlap within the NIR region, its intensity was very weak (Fig. S4), resulting in this nanoconsturct displaying the lowest PA responses among all particles (up to 0.35 ± 0.03 at 200 μM silver). It is worth noting that the PA intensities depend on multiple factors, including the position and intensity of the plasmon bands and absorption coefficients of the particles. Because the experiments were performed at fixed amounts of silver, larger particles, such as 100 nm CB, were present at lower (particle) concentrations, resulting in lower overall extinctions. Furthermore, large particles, such as 100 nm CB, display significantly greater scattering cross sections than absorption cross sections, being less efficient at absorbing incident photons and converting them into heat. Notably, for all five AgNPs, no noticeable loss of PA signal intensity was observed over 10 min continuous irradiations at 705 nm, highlighting the photostability and robustness of the silver probes (Fig. S5). The stability assays for all nanoconstructs were performed under the same exposure conditions for better comparison, and the excitation wavelength of 705 nm was chosen because all nanoparticles displayed (moderated to very strong) PA responses in that wavelength.

**Fig. 4:**
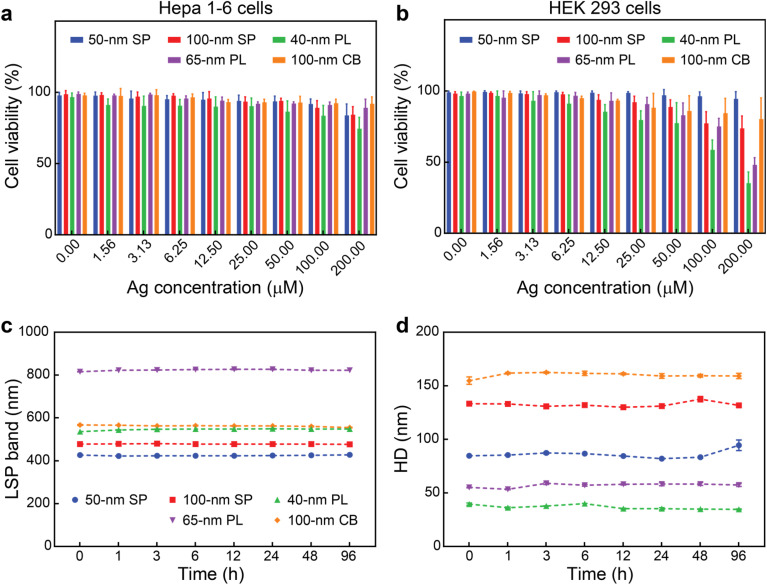
*In vitro* assessment of the AgNPs. Cell viability of (a) Hepa 1–6 cells and (b) HEK 293 cells after 24 h incubations with AgNPs (at 0 to 200 μM silver). (c) LSP band positions and (d) hydrodynamic diameter (HD) of the AgNPs at different incubation times in 10% FBS. Values represent mean ± standard deviation. All measurements were performed in triplicate.

As new silver nanomedicines are progressively moving into clinical trials, and the PVP-coated AgNPs explored in this study are intended to be used as PA imaging probes, we assessed their biocompatibility *in vitro*. Two different cell lines commonly used in toxicological assays, namely mouse hepatoma cell line Hepa1-6 and human embryonic kidney (HEK) 293 cells, were used for the cytotoxicity assessment of the AgNPs. 50 nm SP and 100 nm CB did not show obvious cytotoxicity towards the two cell lines even under high concentrations (up to 200 μM silver) after 24 h (Fig. 4a and b). 100 nm SP and 65 nm PL induced some cytotoxicity (over 20% cell growth inhibition) but only to HEK 293 cells and under high silver concentration (100 and/or 200 μM silver). Among the five nanoformulations, 40 nm PL affected cell viability the most (primarily to HEK 293 cells), as around 20% growth inhibition was observed at 25 μM silver. These results were consistent with previous literature focused on other therapeutic silver nanoconstructs,^59^ which indicated that polymer-functionalized AgNPs were overall not acutely toxic. To further characterize the behavior of the AgNPs in complex biological environments, their stability in 10% fetal bovine serum (FBS) was assessed. Notably, no significant variations were observed in the LSP band positions and hydrodynamic diameters of the particles up to 96 h, indicating that the AgNPs were stable in the complex biological environment (Fig. 4c and d).

**Fig. 5:**
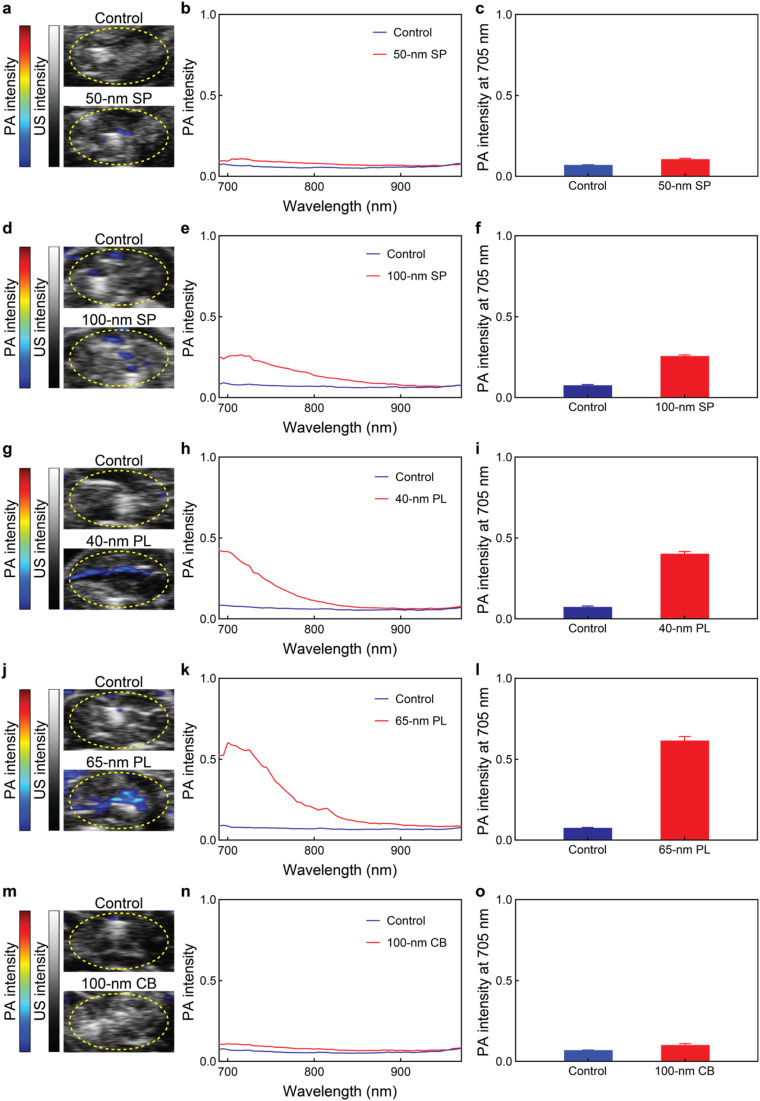
*Ex vivo* PA-US imaging of the AgNPs. PA-US images, PA spectra and PA intensities at 705 nm of (a–c) 50 nm SP, (d–f) 100 nm SP, (g–i) 40 nm PL, (j–l) 65 nm PL, and (m–o) 100 nm CB intramuscularly injected (50 μL, 0 or 200 μM silver) in the legs of mouse cadavers. The scale bar is displayed in linear arbitrary units. The regions of interest for the spectral acquisition are highlighted by yellow dashed areas in (a, d, g, j and m). Values represent mean ± standard deviation. All measurements were performed in triplicate.

Lastly, encouraged by the outstanding PA responses of the AgNPs in gelatin and plastic tube phantoms, and their stability in biological environments, we assessed their PA imaging performance *ex vivo* (Fig. 5). Mouse cadavers from a previous experiment were repurposed for the *ex vivo* imaging, and therefore, no animals were sacrificed for the current study. The different AgNPs (50 μL, 200 μM silver) were intramuscularly injected into the legs of the deceased mice and imaged from 680 to 970 nm with a preclinical PA system. Consistent with the *in vitro* imaging, the 50 nm SP and 100 nm CB showed very weak PA responses (0.11 ± 0.01 and 0.10 ± 0.01, respectively). Meanwhile, the 100 nm SP displayed moderate PA signal intensities (0.26 ± 0.01). Notably, the stronger PA signals of the two nanoplate formulations, 40 nm PL and 65 nm PL, were well preserved in the biological (*ex vivo*) environment (0.40 ± 0.01 and 0.62 ± 0.02, respectively). The 65 nm PL had the strongest PA intensities, as these nanoconstructs were the only ones to display LSP bands centered in the NIR region and their LSP bands were more intense due to their morphology. It is worth noting that the current study focused on the impact of inherent features (*e.g.* shape, extinction, and LSP band position) of silver probes on their PA performance. Nevertheless, other (indirect) *in vivo* characteristics, such as biodistribution, pharmacokinetics, and pathological tissue targeting, are also important in defining the nanoconstruct performances and shall be evaluated in future studies to further demonstrate the advantages of silver nanomedicines over conventional small-molecule imaging agents.

Overall, these results demonstrate that AgNPs can be strong PA probes, and their intensity is strongly affected by their core morphology. Among the different AgNPs explored, the 65 nm PL exhibited the strongest PA contrasts because their LSP band was centered in the NIR and had the highest extinction. The PA responses of AgNPs were well-preserved in complex biological media and *ex vivo* environments. This study paves the way for future *in vivo* research, leveraging the PA properties of AgNPs to gain deeper insights into the endogenous behavior of emerging silver nanomedicines during their (pre)clinical development, as well as broadens their potential applications, including intraoperative imaging and sentinel lymph node mapping.

## Conclusion

We assessed the structural and optical characteristics that define the PA imaging performance of silver nanoconstructs. Five different probes, comprised of a silver (spherical, plate, or cube) core functionalized with a polymeric shell, were explored. The PVP-based polymer coating provided stability in biological environments and preserved the silver nanoconstruct properties after lyophilization. Among the different AgNP cores tested, the 65 nm PL exhibited the strongest PA contrast, because their LSP was centered in NIR and their highest extinction in that region. Furthermore, the PA contrast abilities of our AgNPs were preserved in biological environments, as exemplified by *ex vivo* imaging in deceased mice. Together, our results highlight the potential of silver nanoconsturcts as PA imaging probes, which hold promise for further diagnostic applications, including intraoperative imaging and sentinel lymph node mapping.

## Materials and methods

### Materials

50 nm SP, 100 nm SP, 40 nm PL, 65 nm PL, and 100 nm CB were purchased from nanoComposix (USA). Dulbecco's modified Eagle's medium (DMEM, 4.5 g L^−1^ glucose) and minimum essential medium (MEM) were obtained from Gbico (UK). Fetal bovine serum (FBS) was purchased from Pan-Biotech (Germany). XTT-based cell proliferation kit was purchased from Biological Industries (Israel). Deionized (DI) water was produced by a PURELAB flex 2 device (ELGA LabWater, Germany) and used for all experiments.

### Characterization

An Infinite M200 Pro microplate reader (Tecan, Switzerland) was used to measure the extinction spectra of the AgNPs. The reader was set in absorbance scan mode with a scanning interval of 2 nm. A Nanosizer ZS (Malvern, UK) was used to determine the hydrodynamic diameter and zeta potential. A 100 kV transmission electron microscope system (Hitachi, Japan) was used to image the morphology. The composition of AgNPs was analyzed with a STEM-Detector using a scanning electron microscope (Quattro S, Thermo Fisher) at 30 kV acceleration voltage in a high vacuum. AgNPs were placed on carbon-filmed copper grids by drop-casting before the electron microscope imaging. A Spectrum 3 FTIR spectrometer (PerkinElmer, USA) was used to measure the Fourier-transform infrared (FTIR) spectra. Before FTIR analysis, the AgNPs were lyophilized with an Alpha 2–4 LD PLUS (Martin Chirist, Germany).

### Lyophilization

4 mL of each AgNP solution (200 μM silver) was rapidly frozen by immersing the sample in liquid nitrogen for 1 hour. The samples were then subjected to freeze-drying under vacuum (Alpha 2–4 LD PLUS, Martin Chirist, Germany) for 48 hours. The dried AgNPs were reconstituted with 4 mL of DI water by vigorously vortexing. To evaluate the shelf lives, all AgNPs were left undisturbed for up to 5 weeks after reconstitution. The extinction spectra were normalized for easier LSP band comparison between samples before and after lyophilization.

### Stability in 10% FBS

The AgNPs were dispersed in 10% FBS aqueous solutions and left undisturbed. At each time point (0, 1, 3, 6, 12, 24, 48 and 96 h), their hydrodynamic diameter and extinction spectra were measured.

### PA imaging

The PA imaging of the AgNPs was performed in gelatin phantoms using the VevoLAZR system (Viualsonics, Canada) equipped with an MX250 transducer. The MX250 transducer has an axial resolution of 75 μm and a total imageable area of up to 23 × 30 mm. To make the gelatin phantom, 40 g of gelatin was dissolved in 400 mL of warm DI water (40–50 °C). The 10% (w/v) gelatin solution was poured into the mold and solidified at 4 °C overnight. To acquire the PA spectra, 0.3 mL of each AgNP solution (0, 50, 100, 200 μM silver, 2% (w/v) gelatin) was added into the sample hole (<10 mm × 10 mm) in the 10% (w/v) gelatin phantom, left to solidify for 5 h at 4 °C, and then imaged in the spectra acquisition mode with excitation wavelengths from 680 to 970 nm. The PA stability of the AgNPs was performed in low-density polyethylene tubes, saturated with 0.05 mL of each silver nanoparticle solution (200 μM silver). The PA intensity was recorded under 10 min continuous excitation at the single wavelength (705 nm) mode. All PA measurements were performed in triplicate (in three different samples).

To evaluate the PA intensity, the active signal areas in the PA images were selected as the region of interest (ROI). The system determined the total signal intensity within the ROI by adding up the active pixel values in the ROI and then averaging this sum over the total number of pixels within the same ROI. The resulting value was expressed as PA intensity.

### Cytotoxicity studies

The *in vitro* cytotoxicity test was performed with the human embryonic kidney (HEK) 293 cells and mouse hepatoma cell line Hepa1-6 obtained from the DSMZ-German Collection of Microorganisms and Cell Cultures (Germany). Hepa 1–6 cells were cultured in DMEM (4.5 g L^−1^ glucose) supplemented with 10% FBS and 1% penicillin/streptomycin. HEK 293 cells were cultured in MEM supplemented with 10% FBS and 1% penicillin/streptomycin. Both cell lines with a density of 10 000 cells per well were cultured at 37 °C with 5% CO_2_ for 12 h in a 96-well plate. Subsequently, the cells were exposed to AgNP treatments with different concentrations (0, 3.13, 6.25, 12.50, 25.00, 50.00, 100.00 and 200.00 μM silver) and cultured for 24 h. The standard tetrazolium chloride (XTT) assay was performed to determine cell proliferation.

### *Ex vivo* PA imaging

As a control, PA imaging of the legs of mouse cadavers was performed under the Vevo LAZR system and the PA spectra were acquired with excitation wavelengths from 680 to 970 nm. 50 μL AgNPs (200 μM silver) were intramuscularly injected into the legs and then imaged with the same settings. The deceased mice were repurposed from a previous experiment and therefore, no additional animals were sacrificed. The previous animal experiment had been carried out under the authorization of the German State Office for Nature, Environment, and Consumer Protection (LANUV, Germany) and complied with institutional guidelines, EU Directive 2010/63/EU, and German federal animal protection laws.

## Conflicts of interest

The authors have no relevant affiliations or financial involvement with any organization or entity with a financial interest in or financial conflict with the subject matter or materials discussed in the manuscript.

## Supplementary Material

NA-OLF-D5NA00636H-s001

## Data Availability

The data supporting this article have been included as part of the SI. Size distributions of the silver nanoparticles; energy-dispersive X-ray spectroscopy (EDS) micrographs of the silver nanoparitcles; characterizations of the silver nanoparticles; extinction spectra of the silver nanoconsturcts; PA stability of the silver nanoparticles over time in polyethylene tubes (PDF). See DOI: https://doi.org/10.1039/d5na00636h.
