# Supplementary material for: Structural engineering of silver nanoparticles for enhanced photoacoustic imaging
Source: Nanoscale Adv. 2025 Aug 21;7(19):6110–9. doi: 10.1039/d5na00636h (PMC12368605; doi:10.1039/d5na00636h)
Supplement: NA-007-D5NA00636H-s001 [file NA-007-D5NA00636H-s001.pdf]

Supplementary Information (SI) for Nanoscale Advances.
This journal is © The Royal Society of Chemistry 2025

- SUPPORTING INFORMATION -

Structural Engineering of Silver Nanoparticles for Enhanced Photoacoustic Imaging

Rui Zhang,[1] Manuel Dias,[1,2] Yanchen Li,[1] Stephan Rütten,[3] Fabian Kiessling,[1] Twan Lammers,[1] and Roger M. Pallares[1,*]

[1]Institute for Experimental Molecular Imaging, RWTH Aachen University Hospital, Aachen 52074, Germany

[2] Department of Physics, Faculty of Science, University of Lisbon, 1500-274-Lisboa, Portugal

[3] Electron Microscope Facility, RWTH Aachen University Hospital, Aachen 52074, Germany

[*]Corresponding author: rmoltopallar@ukaachen.de

Table of contents

Table S1. Size distributions of the silver nanoparticles S2

Figure S1. Energy-dispersive X-ray spectroscopy (EDS) micrographs of the silver nanoparticles. S3

Figure S2. Characterization of the silver nanoparticles. S4

Figure S3. The shelf lives of the silver nanoparticles after reconstitution. S5

Figure S4. Extinction spectra of the silver nanoconstructs. S6

Figure S5. PA stability of the silver nanoparticles over time in polyethylene tubes. S7

Table S1. Size distributions of the silver nanoparticles	
	Feret diameter (nm)
50-nm SP	51.1 ± 6.9
100-nm SP	101.2 ± 9.7
40-nm PL	37.8 ± 7.3
65-nm PL	65.8 ± 21.2
100-nm CB	103.3 ± 8.9

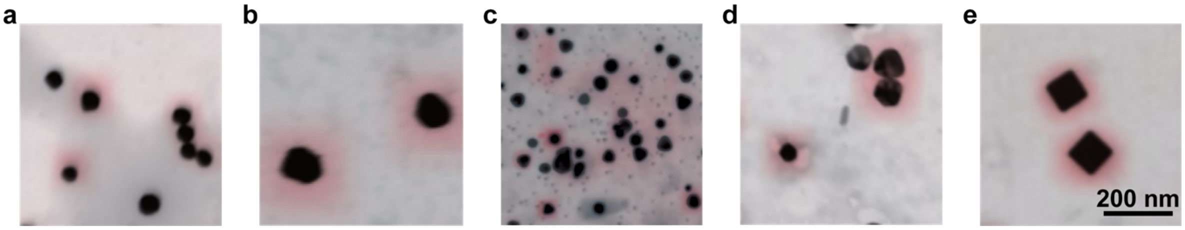

Figure S1. Energy-dispersive X-ray spectroscopy (EDS) micrographs of the silver nanoparticles. EDS micrographs of (**a**) 50-nm SP, (**b**) 100-nm SP, (**c**) 40-nm PL, (**d**) 65-nm PL, and (**e**) 100-nm CB. Signal of silver is displayed in red.

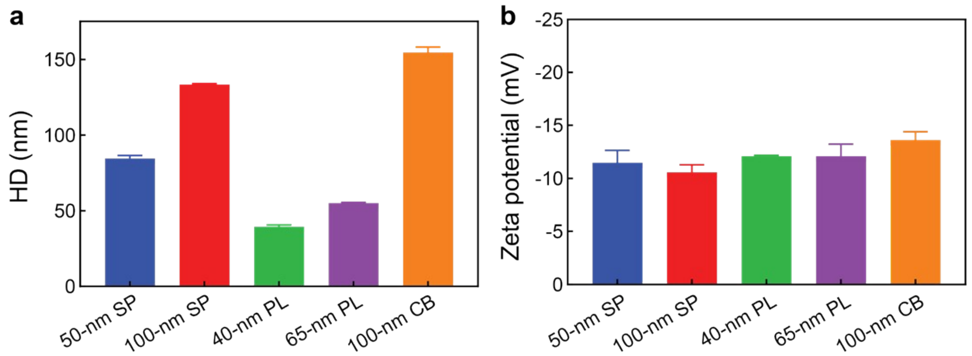

Figure S2. Characterization of the silver nanoparticles. (**a**) Hydrodynamic diameter (HD) and (**b**) zeta potential of silver nanoparticles. Values in columns represent mean ± standard deviation. All measurements were performed in triplicate.

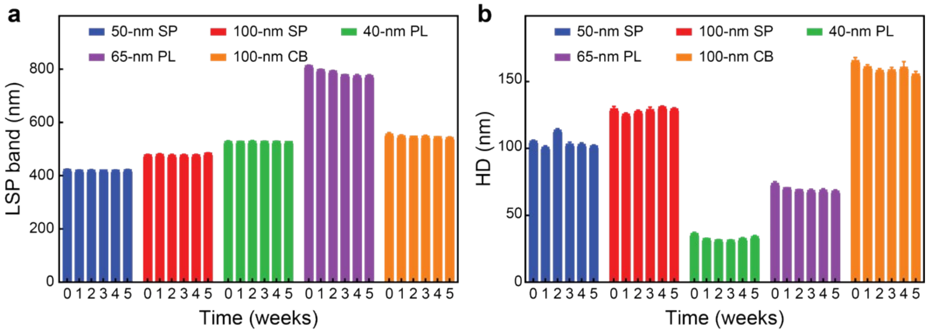

Figure S3. The shelf lives of the silver nanoparticles after reconstitution. (**a**) LSP band positions, and (**b**) hydrodynamic diameters (HD) of the reconstituted silver nanoparticles for five weeks. Values in columns represent mean ± standard deviation. All measurements were performed in triplicate.

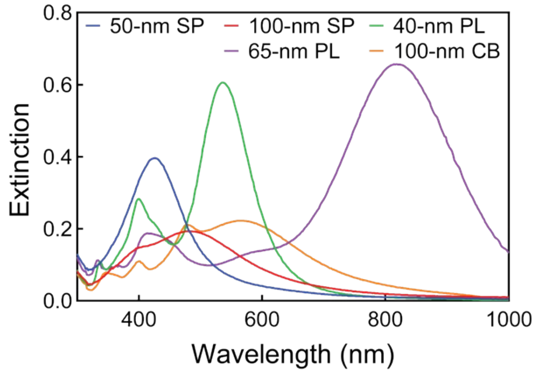

Figure S4. Extinction spectra of the silver nanoconstructs. All measurements were performed at 200 μM silver.

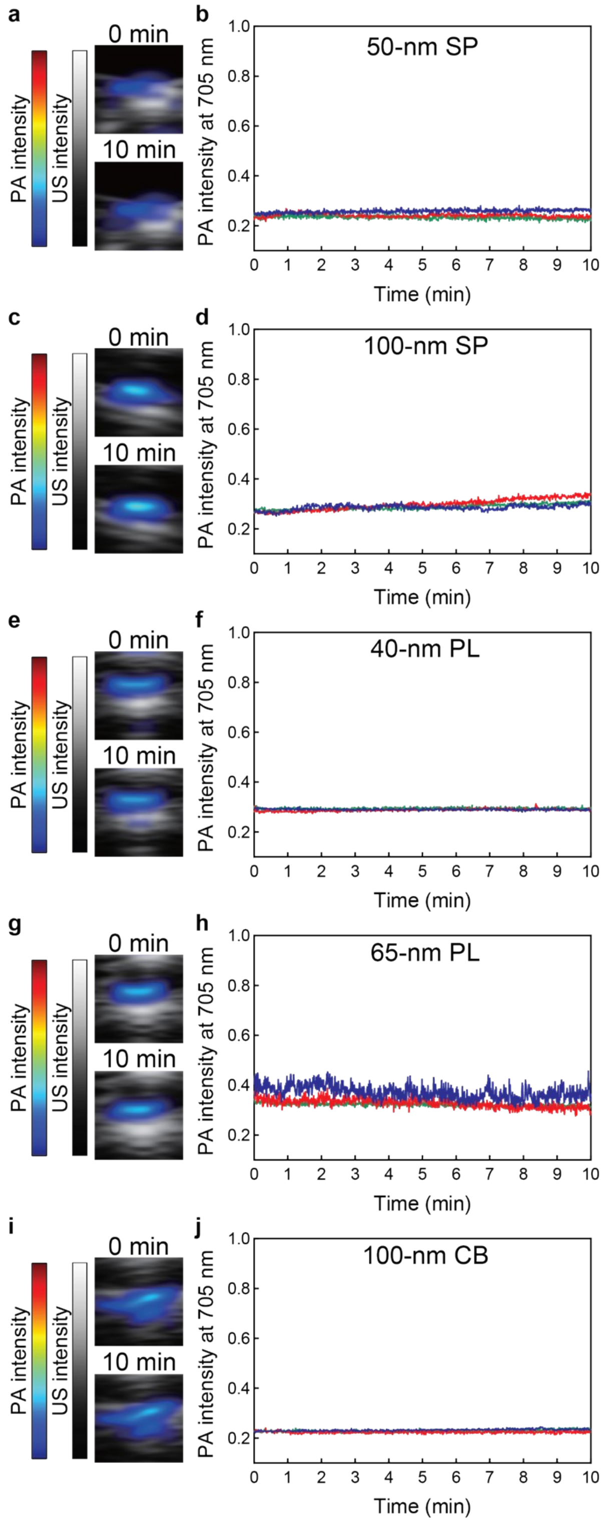

Figure S5. PA stability of the silver nanoparticles over time in polyethylene tubes. PA-US images and PA intensity at 705 nm of (**a**, **b**) 50-nm SP, (**c**, **d**) 100-nm SP, (**e**, **f**) 40-nm PL, (**g, h**) 65-nm PL, and (**i**, **j**) 100-nm CB in polyethylene tubes over 10-min continuous laser irradiation. The scale bar is displayed in linear arbitrary units. All experiments were performed at 200 μM silver and in triplicate (in three different samples).
